# Histological characteristics of advanced peri-implantitis bone defects in humans

**DOI:** 10.1186/s40729-020-00208-8

**Published:** 2020-03-25

**Authors:** Maria Elisa Galárraga-Vinueza, Stefan Tangl, Marco Bianchini, Ricardo Magini, Karina Obreja, Reinhard Gruber, Frank Schwarz

**Affiliations:** 1 Department of Oral Surgery and Implantology, Carolinum, Goethe University, Frankfurt, Germany; 2 Post-Graduate Program in Implant Dentistry (PPGO), Federal University of Santa Catarina (UFSC), Florianópolis, SC Brazil; 3 Core Facility Hard Tissue and Biomaterial Research, Karl Donath Laboratory, University Clinic of Dentistry, Medical University of Vienna, Vienna, Austria; 4 Austrian Cluster for Tissue Regeneration, Vienna, Austria; 5 Department of Oral Biology, University Clinic of Dentistry, Medical University of Vienna, Sensengasse 2a, Vienna, Austria

**Keywords:** Case study, Peri-implantitis, Histomorphometry

## Abstract

**Background:**

Inflammatory osteolysis is the clinical hallmark of peri-implantitis. The morphology of the remaining peri-implant bone and the level of osseointegration, however, remain unknown. Our aim was to characterize advanced peri-implantitis bone defects in humans.

**Methods:**

Four patients (3 female and 1 male) were diagnosed with peri-implantitis. A total of 5 implants with machined surfaces and a mean loading time of 12 ± 6 years were removed due to advanced bone loss. The defect extension, the peri-implant bone density (bone area per tissue area in percentage), bone-to-implant contact (%), and the number of filled and empty osteocyte lacunae were calculated based on undecalcified histological specimens.

**Results:**

The defect extension was on average 4.2 mm (95% CI 0.8–3.4). Remaining peri-implant bone showed a high density of 85.5% (95% CI 79.1–91.3) and covered in total 74% (95% CI 70.5–77.5) of the implant surface. Filled and empty osteocyte lacunae density was on average 191 and 165/mm^2^ (95% CI 132–251; 103–225), respectively. Histology further revealed signs of ongoing bone formation and resorption.

**Conclusion:**

There are signs that suggest that once the original cortical bone is lost due to peri-implantitis, the remaining apical trabecular bone is reinforced and transformed into cortical bone that might take over the functional load.

## Background

Peri-implantitis was recently defined as a “pathological condition occurring in tissues around dental implants”, which is “characterized by inflammation in the peri-implant connective tissue and a progressive loss of supporting bone” [1–4].

Human biopsies confirmed the presence of plasma cells, macrophages, and neutrophils [5] and elevated expression of inflammatory cytokines [6, 7] at peri-implantitis sites. Moreover, bone matrix molecules were decreased and cells had a fibroblastic phenotype [8]. Together, human biopsies of the respective soft tissue revealed the conserved cellular and molecular principles of acute inflammatory osteolysis [9].

Peri-implantitis lesions extend apical and are commonly associated with a circumferential pattern of bone loss [1, 10]. Intraoperative clinical and radiographic assessments, however, do not fully represent the extension of peri-implantitis defects [11–14]. Moreover, the morphology of the remaining bone being exposed to masticatory forces has not been described so far. Considering that the remaining peri-implant bone has to adapt to occlusal load [15], it requires vital osteocytes to translate mechanical forces into molecular signals controlling bone adaptation [16]. It also requires vital osteocytes for inflammatory osteolysis [17]. These biological principles let us to hypothesize that the bone in peri-implantitis defects has a high density and contains vital osteocytes. Therefore, the present study reports on the characteristics of bone at sites of advanced peri-implantitis.

## Materials and methods

### Subjects and implant data

The present study included 4 patients, 3 female and 1 male (mean age of 66 ± 5 years), who attended the Implant Dentistry Department at Federal University of Santa Catarina, complaining of pain, discomfort, suppuration, and/or bleeding at implant sites. All patients revealed a history of periodontal disease, were non-smokers (100%), and reported no additional systemic diseases. The patients exhibited a total of 5 machined titanium implants with a mean loading time of 12 ± 6 years, supporting single screw-retained prostheses and were diagnosed with peri-implantitis.

Peri-implantitis was defined as the combination of bleeding on gentle probing (BOP) with or without suppuration, probing depths (PD) ≥ 6 mm, and radiographic marginal bone loss (MBL) (i.e., interproximal bone levels ≥ 3 mm apical of the most coronal portion of the intraosseous part of the implant) [2]. All implant sites were associated with an advanced bone loss (> 6 mm/> 50% of the implant length) [14] and were clinically stable (i.e., no manually detected mobility) but were scheduled for explantation due to the advanced disease progression. The study protocol no. 3437751 was approved by the Human Research Committee at Federal University of Santa Catarina-Brazil, 2016–2021, and all patients signed an informed consent according to the university ethics regulations.

### Clinical evaluation

The following clinical measurements were recorded using a color-coded plastic periodontal probe (PCV12PT Hu-Friedy Inc., Chicago, IL, USA): (1) BOP, evaluated as present if bleeding was evident within 30 s after probing, or absent, if no bleeding was noticed within 30 s after probing; (2) PD measured from the mucosal margin to the bottom of the pocket; (3) suppuration, evaluated as present if it was evident after probing and/or peri-implant palpation. All measurements were performed at 6 aspects per implant: mesio-vestibular (mb), mid-vestibular (b), disto-vestibular (db), mesio-oral (mo), mid-oral (o), and disto-oral (do) by one calibrated investigator (M.B.).

### Radiological evaluation

Cone beam computed tomographic (CBCT) scans were obtained from each patient for the surgical planning of implant removal. Linear measurements of the defect length (DL) at affected implants were made by drawing a vertical line, following the long axis of the implant, from the implant shoulder (IS) to the bottom of the defect (BD) at buccal and oral aspects in the CBCT data sets (Implant viewer, version 1.84, USA). Radiological evaluations were performed by one experienced and calibrated examiner (M.G.).

### Surgical procedure and retrieval of specimens

Implant removal in all patients was performed by the same clinician (M.B.) following a standardized surgical procedure. Patients were prescribed 500 mg of oral amoxicillin, 3 times a day during 1 week, starting 1 day before surgery. Patients were locally anesthetized (2% lidocaine, 1:100,000 epinephrine). The prostheses of the implants were removed. Surgical incision was performed, and full-thickness mucoperiosteal lingual and buccal flaps were raised; bone-implant biopsies were obtained using a trephine bur under copious irrigation with sterile saline. The implants were removed with the minimal required trephine diameter and without compromising the soft-tissue component for ethical reasons. Specimens were immersed immediately in 10% neutral buffered formalin. Following the removal of granulation tissue from the defect area, mucoperiosteal buccal and lingual flaps were repositioned and fixed with single sutures.

### Histological processing

Specimens were dehydrated in ascending series of alcohol and embedded in light-cured resin (Technovit 7200, VLC+BPO, Heraeus, Kulzer Co., Wehrheim Germany). Undecalcified thin ground sections were prepared along the longitudinal axis (bucco-lingual direction) with a high precision diamond-coated band saw and precision grinding equipment (Exakt, Apparatebau, Norderstedt, Germany) [18]. Histological sections were stained with Levai-Laczko dye and scanned with an Olympus BX61VS microscope using a digital virtual light microscopy system (dotSlide 2.4; Olympus, Tokyo, Japan) at a resolution of 0.321 μm/pixel.

### Histomorphometrical analysis

**Fig. 1:**
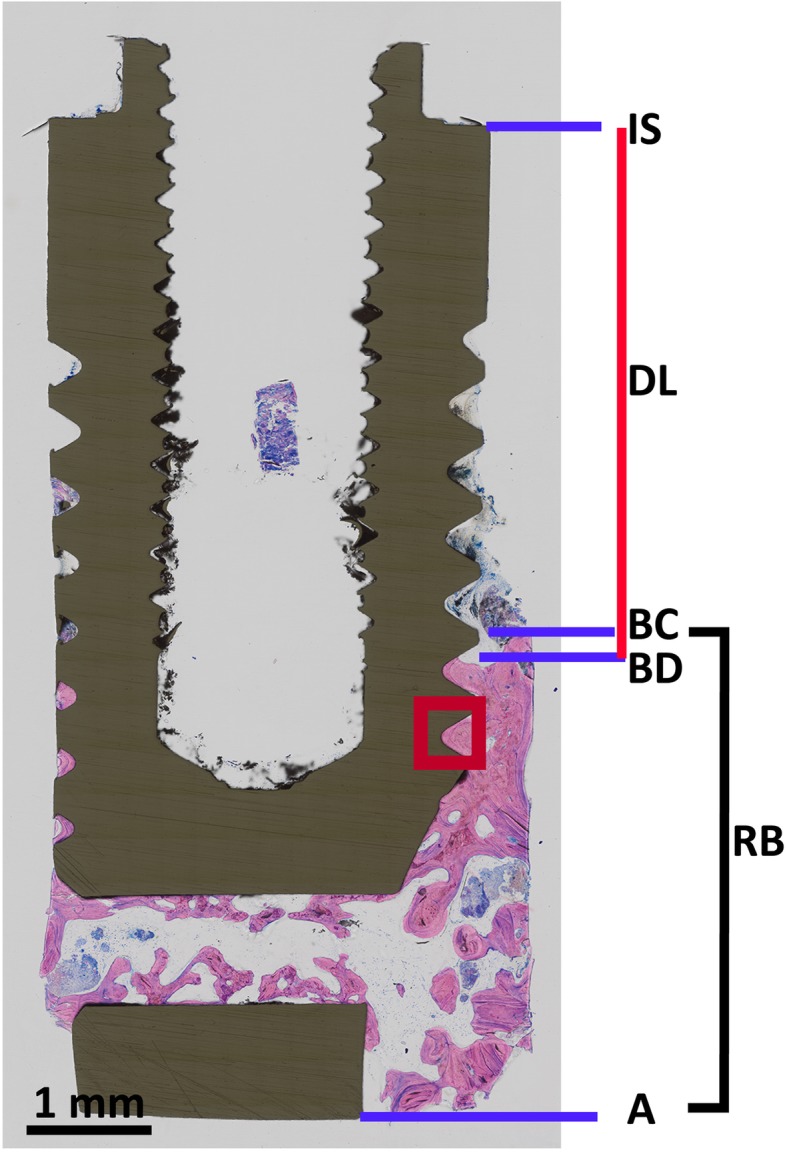
Histological section illustrating the landmarks to determine the conducted histomorphometrical length measurements: DL, RB, and BIC at buccal RB. The red square frames the intra-thread area (ROI) for OD and ELD analysis

Histomorphometrical analysis and microscopic observations were performed by one calibrated examiner (M.G.). Digitalized histological sections (bucco-lingual direction) were analyzed using a morphometric software (Definiens Developer XD 2.0, Definiens AG, Munich Germany). The following landmarks were identified in the stained sections (buccal aspect) (Fig. 1): implant shoulder (IS), level of the alveolar bone crest (BC), bottom of the defect (BD), and the most apical extension of the residual bone tissue (A). Linear measurements were performed by drawing a vertical line following the long axis of the implant: defect length (DL), as measured from IS to BD (mm), residual bone (RB) as measured from BC to A, residual BIC (i.e., the length proportion of the implant surface that was in direct contact with mineralized tissue) as measured as a percentage of the distance from BD to A. The density of the RB was measured as a percentage by dividing the bone area (B.Ar) by the total area of tissues (T.Ar) surrounding the implant.

**Fig. 2:**
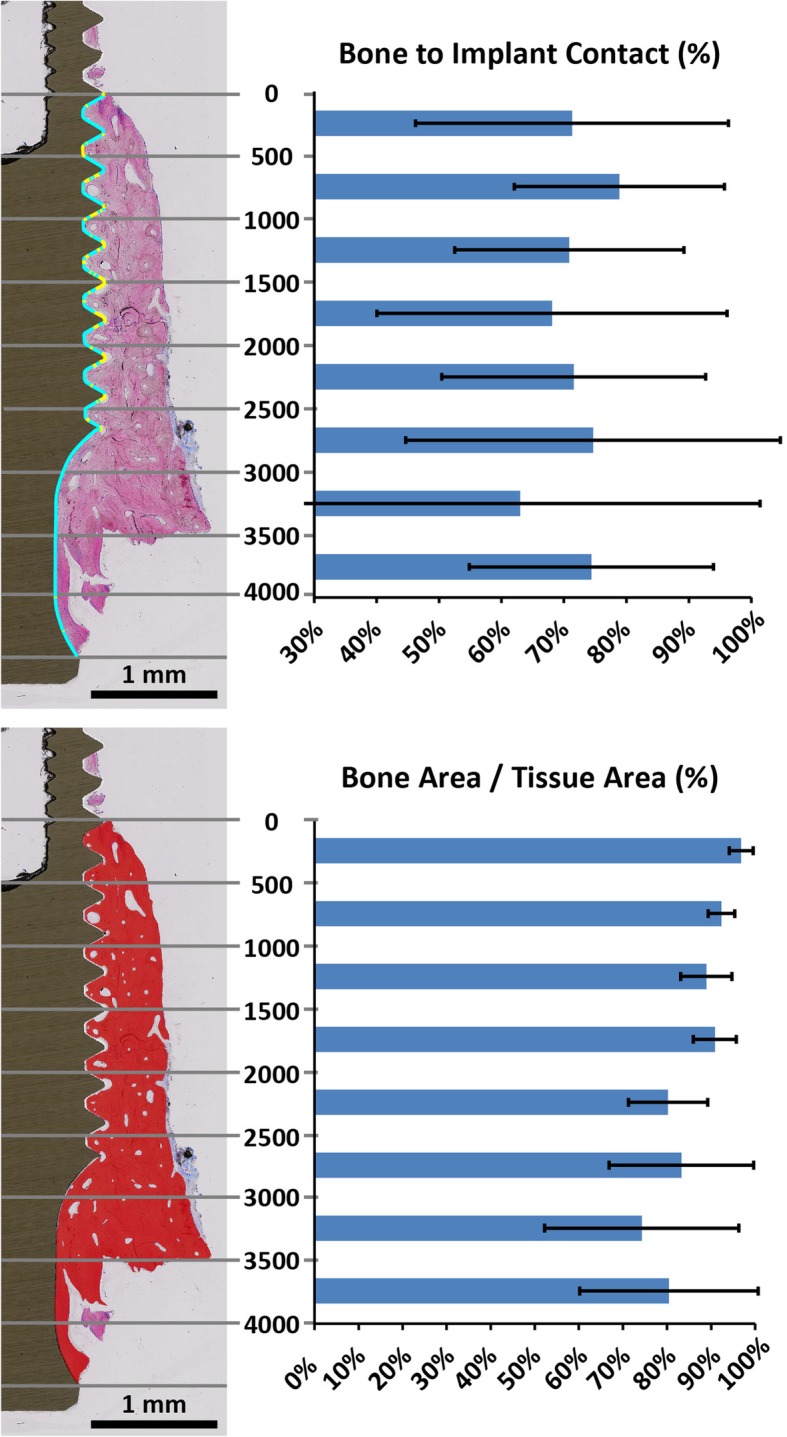
Histological view (buccal aspect) displaying mean BIC (%) and mean bone density (%) at corresponding regions (500 μm zones) from BD to A. Blue lines along the implant perimeter display BIC presence and yellow lines display BIC absence

RB was divided into 500-μm-wide horizontal zones (from BD to A) following the long axis of the implant. For each of these individual zones, BIC and bone density (B.Ar/T.Ar) were determined. Osteocyte and empty osteocytic lacunae were counted along the region of interest (ROI) (i.e., the area encompassing the intra-thread regions along the buccal RB extension) (Fig. 1) using a software program (Image J, software, 1.52a, Maryland, USA). Osteocyte density and empty lacunae density were determined by calculating the ratio of the number of osteocytes or empty lacunae in the region of interest.

### Statistical analysis

The statistical analysis was performed using a commercially available software program (SPSS, 19.0, Chicago, IL, USA). Mean values, standard deviations, and confidence intervals for each variable were calculated at the implant level.

## Results

### Clinical and radiographic measurements

The clinical and radiographic characteristics of the implant sites are presented in Table 1. In total, 3 implants were located in the maxilla, while two implants were located in the mandible. While all implants showed bleeding on probing, only 2 implants presented with suppuration on gentle probing. Mean probing depth was 6.3 ± 0.4 mm corresponding to a mean marginal bone loss (MBL) of 4.7 ± 1.0 mm at the buccal and 7.6 ± 1.3 mm at the palatal/lingual aspects.

**Table 1:** Patient and implant site characteristics

Sample ID	Gender	Age	Location	Prosthesis type	Loading time (years)	lmplant surface	Mean BOP (%)	Supp (+/−)	Mean PD (mm)	MBL (mm)
1	M	69	Maxilla	Single-screwed	5.1	Machined	100	+	6.4	B: 4.0 P: 6.0
2	F	60	Mandible	Single-screwed	10.0	Machined	100	**−**	6.0	B: 4.5 L: 5.0
3	F	60	Mandible	Single-screwed	10.0	Machined	100	**−**	6.2	B: 4.0 L: 5.0
4	F	70	Maxilla	Single-screwed	20.3	Machined	100	**−**	7.0	B: 4.7 P: 8.0
5	F	69	Maxilla	Single-screwed	15.0	Machined	100	+	6.0	B: 4.0 P: 7.0

*B* buccal aspect, *P* palatal aspect, *L* lingual aspect, *Supp* suppuration, *BOP%* bleeding on probing score, *MBL* marginal bone loss, *PD* probing depth, + presence, − absence

### Histomorphometric measurements

Defect length, residual bone extension, bone density, as well as BIC values are presented in Table 2. The mean defect length amounted to 4.7 mm (95% CI 3.12–4.88). Bone density (B.Ar/T.Ar) as exemplified in Fig. 2 was 85.5% (95% CI 79.1–91.3) in the peri-implant tissue thereby indicating a strong corticalization. The implants revealed a mean bone-to-implant contact of 74% (95% CI 70.5–77.5), not reduced at marginal regions (0–500 μm segment) when compared with the more apical regions (Fig. 2). Osteocyte lacunae with signs of stained osteocytes and such with no viable cells exhibited mean values of 191.2/mm^2^ (95% CI 132–251) and 164.6/mm^2^ (95% CI 103–225), respectively (Table 3).

**Table 2:** Results from histomorphometric measurements exhibiting DL, RB, bone density (%), residual bone-to-implant contact (%) values

Specimen ID	DL (mm)	RB (mm)	B.Ar/T.Ar (%)	BIC (%)
1	4.8	3.5	72.4	80.0
2	5.6	8.9	92.3	75.0
3	5.6	5.3	88.1	73.8
4	3.8	7.7	81.8	68.7
5	3.6	3.2	92.5	71.1
Mean	4.7	5.7	85.5	74.0
SD ±	1.0	2.5	8.0	4.0
95% CI	3.1–4.9	3.3–6.8	78–92	70.6–77.6

*SD* standard deviation, *CI* confidence interval, *SA* Surface area

**Fig. 3:**
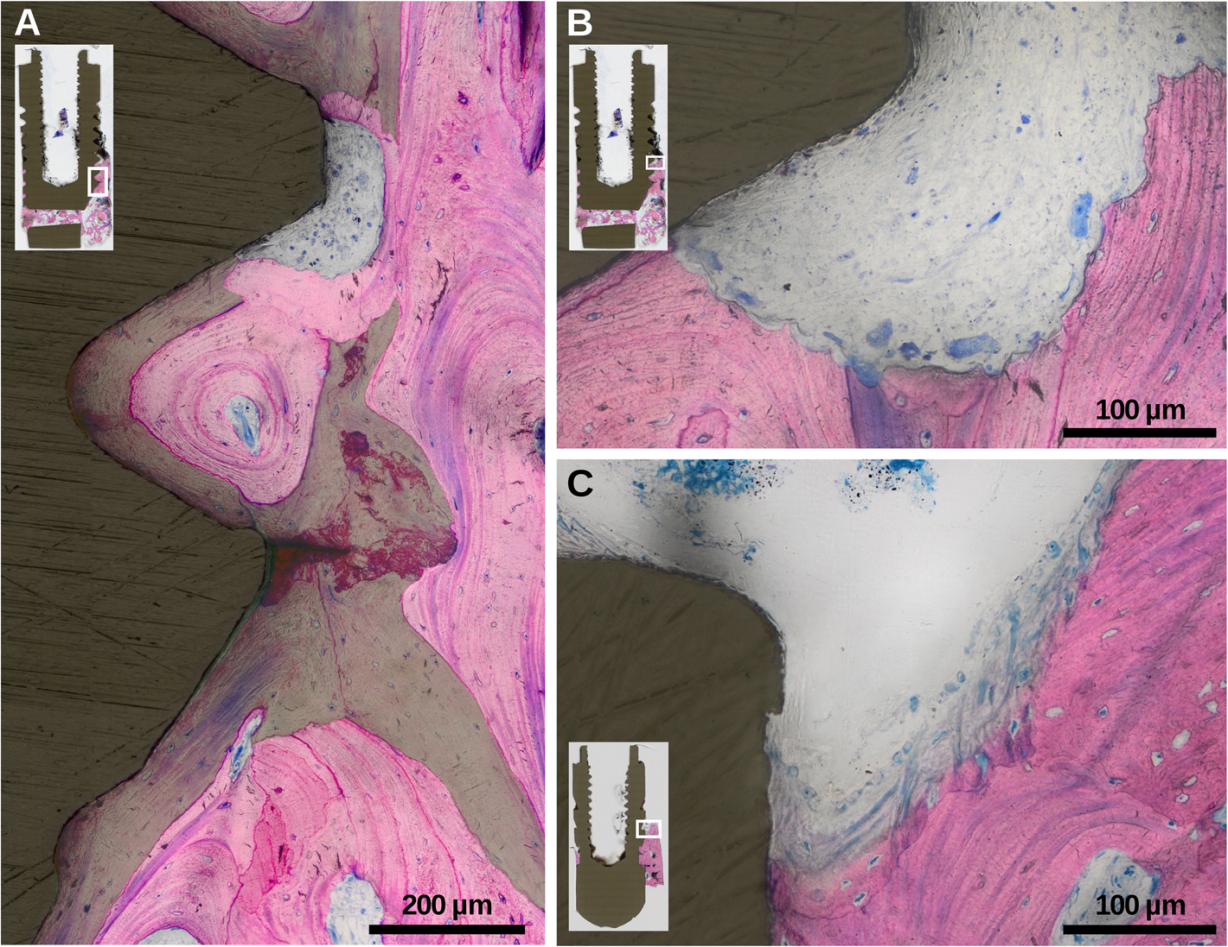
Histological magnifications of specimens exposing **a** significant areas of lamellar bone in-between and parallel to the surface of older trabeculae (accentuated by green overlay), **b** osteoclastic activity, and **c** active bone formation at intrabony marginal regions

**Table 3:** Mean osteocyte (OD) and empty lacunae density (ELD) at peri-implant residual bone

SP ID	Mean OD (number/mm^2^)	Mean ELD (number/mm^2^)	Osteocyte/empty lacuna ratio
1	293	231	1.26
2	221	102	1.32
3	112	245	0.45
4	172	102	1.68
5	162	143	1.12
Mean	192	165	1.17
SD ±	68	69.2	0.45
95% CI	(132–251)	(103–225)	

*SD* standard deviation

### Histological observations

Two specimens revealed a mixed chronic inflammatory cell infiltrate in the marginal connective tissue compartments adjacent to the bone surface. One specimen showed signs of osteoclastic activity (Fig. 3b), 3 specimens presented inactive crestal bone surfaces, and 1 specimen showed active bone formation (Fig. 3c) at marginal regions. According to the histomorphometric data, the residual bone was predominantly cortical with scarce and small-sized vascular channels. Importantly, the cortical bone exhibited areas of lamellar bone in-between and parallel to the surface of older trabeculae, depicting signs of compaction of former cancellous bone (Fig. 3a). Secondary osteons and reversal lines were frequently present, while osteoclasts were found only occasionally. Cancellous bone was merely present at the most apical region of two specimens.

## Discussion

The present study was inspired by the concept that upon advanced loss of cortical peri-implant bone, the remaining peri-implant trabecular bone is subjected to masticatory forces and reinforced as a consequence of functional adaptation [15]. This adaptation depends on vital osteocytes [16]. Therefore, the present human case series aimed at investigate the peri-implant bone in advanced peri-implantitis with a focus on the morphology and the vitality of osteocytes. The histomorphometric analysis suggests that bone remaining at advanced peri-implantitis is vital and mainly cortical, presumably originating by reinforcement of the original trabecular bone.

Previous studies also used human biopsy material, but focused on the histopathological features of soft-tissue samples taken at peri-implantitis sites [5, 19, 20]. So far, disease progression and related extension of the bony defects have been assessed histologically in preclinical ligature models resulting in 5 to 7 mm peri-implant defects within 1 year [21–23]. None of the aforementioned preclinical studies reported a histomorphometric analysis of the residual bone, and the implants were not subjected to masticatory loading. Thus, we cannot relate our findings to those observed with previous preclinical studies except with respect to the extent of advanced peri-implant defects.

Among the limitations of the present study is the lack of soft tissue in the biopsy due to ethical considerations. Accordingly, the histological outcomes could not be correlated with inflammatory infiltrates residing in the subepithelial connective tissue compartment [6, 7, 20]. It also remains unclear to what extent the progression of bone loss may even support osseointegration of the implants. Previous studies evaluating human biopsy material have reported a wide range of bone-to-implant contacts ranging from 19 to 93% at machined implants [24–26]. The high bony coverage of these and our cases can be explained by the functional corticalization of the originally trabecular bone. Strong support for this hypothesis comes from our histological observations where marginal bone showed characteristic signs of heterogeneous bone remodeling with significant areas of lamellar bone in-between and parallel to the surface of older trabeculae, representing signs of compaction of former trabecular bone, an observation also made, e.g., during the growth of the metaphysis [27].

When further interpreting the present study, the peri-implant osteocyte lacunae density after 1 to 27 years in function was reported [28]. In line with our findings, empty osteocytic lacunae adjacent to zirconia implants diagnosed with peri-implantitis were described [29]. Based on counting the osteocyte lacunae, we observed that numerous filled but also empty osteocytic lacunae were present within the residual bone. To which extend the osteocytes in the remaining peri-implant bone control bone remodeling [30] , the adaptation to functional loading termed modeling [16], and also the progression of inflammatory osteolysis [17] is unknown and opens pathways for future research.

## Conclusions

In conclusion, the findings of this case series based on dental implants that had to be retrieved because of advanced peri-implantitis and the concomitant inflammatory osteolysis suggest that the residual peri-implant bone might accumulate the functional load and is consequently modeled and reinforced to become cortical bone.

## Data Availability

The data sets used and/or analyzed during the current study are available from the corresponding author or reasonable request.

## Abbreviations

A: Most apical extension of the residual bone tissue
B.Ar: Bone area
BD: Bone defect
BIC: Bone-to-implant contact
BOP: Bleeding on probing
CBCT: Cone beam computed tomography
CI: Confidence interval
db: Disto-buccal
DL: Defect length
do: Disto-oral
ELD: Empty lacuna density
IS: Implant shoulder
mb: Mesio-buccal
MBL: Marginal bone loss
mo: Mesio-oral
o: Oral
OD: Osteocyte density
PD: Probing depth
RB: Residual bone
ROI: Region of interest
SA: Surface area
SD: Standard deviation
T.Ar: Total area of tissues

## References

[1] Schwarz F, Derks J, Monje A, Wang H-L (2018). Peri-implantitis. J Clin Periodontol..

[2] Berglundh T, Armitage G, Araujo MG, Avila-Ortiz G, Blanco J, Camargo PM, et al. Peri-implant diseases and conditions: consensus report of workgroup 4 of the 2017 World Workshop on the classification of periodontal and peri-implant diseases and conditions. J Clin Periodontol [Internet]. 2018 [cited 2018 Nov 19];45:S286–S291. Available from: http://www.ncbi.nlm.nih.gov/pubmed/29926491. 10.1111/jcpe.12957 29926491

[3] Dalago HR, Schuldt Filho G, Rodrigues MAP, Renvert S, Bianchini MA. Risk indicators for peri-implantitis. A cross-sectional study with 916 implants. Clin Oral Implants Res [Internet]. 2016 Jan [cited 2016 Jul 15];n/a-n/a. Available from: http://doi.wiley.com/10.1111/clr.12772. 10.1111/clr.12772 26754342

[4] Bianchini MA, Galarraga-Vinueza ME, Apaza-Bedoya K, De Souza JM, Magini R, Schwarz F. Two to six-year disease resolution and marginal bone stability rates of a modified resective-implantoplasty therapy in 32 peri-implantitis cases. Clin Implant Dent Relat Res [Internet]. 2019 [cited 2019 Aug 8];cid.12773. Available from: http://www.ncbi.nlm.nih.gov/pubmed/30985073. 10.1111/cid.12773 30985073

[5] Carcuac O., Berglundh T. (2014). Composition of Human Peri-implantitis and Periodontitis Lesions. Journal of Dental Research.

[6] Jung S-R, Bashutski JD, Jandali R, Prasad H, Rohrer M, Wang H-L. Histological analysis of soft and hard tissues in a periimplantitis lesion. Implant Dent [Internet]. 2012 [cited 2019 Mar 13];21(3):186–189. Available from: https://insights.ovid.com/crossref?an=00008505-201206000-00006. 10.1097/ID.0b013e31824eec0a 22526142

[7] Konttinen YT, Lappalainen R, Laine P, Kitti U, Santavirta S, Teronen O. Immunohistochemical evaluation of inflammatory mediators in failing implants. Int J Periodontics Restorative Dent [Internet]. 2006 [cited 2019 Mar 13];26(2):135–141. Available from: http://www.ncbi.nlm.nih.gov/pubmed/16642902. 16642902

[8] Schminke B., vom Orde F., Gruber R., Schliephake H., Bürgers R., Miosge N. (2014). The Pathology of Bone Tissue during Peri-Implantitis. Journal of Dental Research.

[9] Gruber Reinhard (2019). Osteoimmunology: Inflammatory osteolysis and regeneration of the alveolar bone. Journal of Clinical Periodontology.

[10] Derks J, Schaller D, Håkansson J, Wennström JL, Tomasi C, Berglundh T. Peri-implantitis - onset and pattern of progression. J Clin Periodontol [Internet]. 2016 [cited 2019 Mar 13];43(4):383–388. Available from: http://doi.wiley.com/10.1111/jcpe.12535. 10.1111/jcpe.12535 26900869

[11] Schwarz F, Herten M, Sager M, Bieling K, Sculean A, Becker J. Comparison of naturally occurring and ligature-induced peri-implantitis bone defects in humans and dogs. Clin Oral Implants Res [Internet]. 2007 [cited 2019 Mar 18];18(2):161–170. Available from: http://doi.wiley.com/10.1111/j.1600-0501.2006.01320.x. 10.1111/j.1600-0501.2006.01320.x 17348880

[12] Serino G, Turri A, Lang NP. Probing at implants with peri-implantitis and its relation to clinical peri-implant bone loss. Clin Oral Implants Res [Internet]. 2013 [cited 2019 Mar 18];24(1):91–95. Available from: http://doi.wiley.com/10.1111/j.1600-0501.2012.02470.x. 10.1111/j.1600-0501.2012.02470.x 22462625

[13] García-García Marta, Mir-Mari Javier, Benic Goran I., Figueiredo Rui, Valmaseda-Castellón Eduard (2016). Accuracy of periapical radiography in assessing bone level in implants affected by peri-implantitis: a cross-sectional study. Journal of Clinical Periodontology.

[14] Monje A, Pons R, Insua A, Nart J, Wang H, Schwarz F. Morphology and severity of peri-implantitis bone defects. Clin Implant Dent Relat Res [Internet]. 2019 [cited 2019 Jul 7];cid.12791. Available from: https://onlinelibrary.wiley.com/doi/abs/10.1111/cid.12791. 10.1111/cid.12791 31087457

[15] Frost Michelle L, Fogelman Ignac, Blake Glen M, Marsden Paul K, Cook Gary JR (2004). Dissociation Between Global Markers of Bone Formation and Direct Measurement of Spinal Bone Formation in Osteoporosis. Journal of Bone and Mineral Research.

[16] Uda Y, Azab E, Sun N, Shi C, Pajevic PD. Osteocyte mechanobiology. Curr Osteoporos Rep [Internet]. 2017 [cited 2020 Jan 8];15(4):318–25. Available from: http://www.ncbi.nlm.nih.gov/pubmed/28612339. 10.1007/s11914-017-0373-0 PMC5656287 28612339

[17] Graves DT, Alshabab A, Albiero ML, Mattos M, Corrêa JD, Chen S, et al. Osteocytes play an important role in experimental periodontitis in healthy and diabetic mice through expression of RANKL. J Clin Periodontol [Internet]. 2018 [cited 2020 Jan 8];45(3):285–292. Available from: http://www.ncbi.nlm.nih.gov/pubmed/29220094. 10.1111/jcpe.12851 PMC5811370 29220094

[18] Donath K. The diagnostic value of the new method for the study of undecalcified bones and teeth with attached soft tissue, (Säge-Schliff, (Sawing and Grinding) Technique). Pathol - Res Pract [Internet]. 1985 1 [cited 2019 Mar 21];179(6):631–633. Available from: https://www.sciencedirect.com/science/article/pii/S0344033885802090. 10.1016/S0344-0338(85)80209-0 3895202

[19] Berglundh T, Zitzmann NU, Donati M. Are peri-implantitis lesions different from periodontitis lesions? J Clin Periodontol [Internet]. 2011 [cited 2019 Mar 13];38:188–202. Available from: http://doi.wiley.com/10.1111/j.1600-051X.2010.01672.x. 10.1111/j.1600-051X.2010.01672.x 21323715

[20] Berglundh T, Gislason O, Lekholm U, Sennerby L, Lindhe J. Histopathological observations of human periimplantitis lesions. J Clin Periodontol [Internet]. 2004 [cited 2019 Mar 13];31(5):341–347. Available from: http://doi.wiley.com/10.1111/j.1600-051X.2004.00486.x. 10.1111/j.1600-051X.2004.00486.x 15086615

[21] Zitzmann NU, Berglundh T, Ericsson I, Lindhe J. Spontaneous progression of experimentally induced periimplantitis. J Clin Periodontol [Internet]. 2004 [cited 2019 Jul 27];31(10):845–849. Available from: http://doi.wiley.com/10.1111/j.1600-051X.2004.00567.x. 10.1111/j.1600-051X.2004.00567.x 15367187

[22] Albouy Jean-Pierre, Abrahamsson Ingemar, Persson Leif G., Berglundh Tord (2009). Spontaneous progression of ligatured induced peri-implantitis at implants with different surface characteristics. An experimental study in dogs II: histological observations. Clinical Oral Implants Research.

[23] Berglundh T., Gotfredsen K., Zitzmann N. U., Lang N. P., Lindhe J. (2007). Spontaneous progression of ligature induced peri-implantitis at implants with different surface roughness: an experimental study in dogs. Clinical Oral Implants Research.

[24] Grassi S, Piattelli A, Ferrari DS, Figueiredo LC, Feres M, Iezzi G, et al. Histologic evaluation of human bone integration on machined and sandblasted acid-etched titanium surfaces in type IV bone. J Oral Implantol [Internet]. 2007 [cited 2019 Sep 10];33(1):8–12. Available from: http://www.joionline.org/doi/abs/10.1563/0-791.1. 10.1563/0-791.1 17410906

[25] Trisi Paolo, Lazzara Richard, Rebaudi Alberto, Rao Walter, Testori Tiziano, Porter Stephan S. (2003). Bone-Implant Contact on Machined and Dual Acid-Etched Surfaces After 2 Months of Healing in the Human Maxilla. Journal of Periodontology.

[26] Iezzi G, Vantaggiato G, Shibli JA, Fiera E, Falco A, Piattelli A, et al. Machined and sandblasted human dental implants retrieved after 5 years: a histologic and histomorphometric analysis of three cases. Quintessence Int [Internet]. 2012 [cited 2019 Sep 10];43(4):287–292. Available from: http://www.ncbi.nlm.nih.gov/pubmed/22532942. 22532942

[27] Enlow DH. A study of the post-natal growth and remodeling of bone’ [Internet]. 1962 [cited 2019 Sep 20]. Available from: https://deepblue.lib.umich.edu/bitstream/handle/2027.42/49627/1001100202_ftp.pdf;sequence=1. 10.1002/aja.1001100202 13890322

[28] Piattelli Adriano, Artese Luciano, Penitente Enrico, Iaculli Flavia, Degidi Marco, Mangano Carlo, Shibli Jamil Awad, Coelho Paulo G, Perrotti Vittoria, Iezzi Giovanna (2013). Osteocyte density in the peri-implant bone of implants retrieved after different time periods (4 weeks to 27 years). Journal of Biomedical Materials Research Part B: Applied Biomaterials.

[29] Kohal Ralf-Joachim, Patzelt Sebastian B. M., Butz Frank, Sahlin Herman (2013). One-piece zirconia oral implants: one-year results from a prospective case series. 2. Three-unit fixed dental prosthesis (FDP) reconstruction. Journal of Clinical Periodontology.

[30] Dallas SL, Prideaux M, Bonewald LF. The osteocyte: an endocrine cell ... and more. Endocr Rev [Internet]. 2013 [cited 2020 Jan 8];34(5):658–690. Available from: https://academic.oup.com/edrv/article/34/5/658/2354663. 10.1210/er.2012-1026 PMC3785641 23612223

